# Performance evaluation of a novel multi-pinhole collimator on triple-NaI-detector SPECT/CT for dedicated myocardial imaging

**DOI:** 10.1186/s40658-023-00541-y

**Published:** 2023-03-25

**Authors:** Aron K. Krizsan, Kornel Kukuts, Walid Al-Muhanna, Zoltan Szoboszlai, Laszlo Balazs, Balazs Szabo, Janos Kiss, Stephan Nekolla, Sandor Barna, Ildiko Garai, Tamas Bukki, Attila Forgacs

**Affiliations:** 1 ScanoMed Nuclear Medicine Centers, Nagyerdei Krt. 98, Debrecen, 4032 Hungary; 2 Mediso Ltd., Budapest, Hungary; 3 Medical Imaging Clinic - Radiology, Clinical Center, University of Debrecen, Debrecen, Hungary; 4 Nuklearmedizinische Klinik und Poliklinik, Klinikum rechts der Isar der Technische Universitӓt München, München, Germany; 5 DZHK (German Centre for Cardiovascular Research), Partner Site Munich Heart Alliance, Munich, Germany; 6 Department of Medical Imaging, Division of Nuclear Medicine and Translational Imaging, Faculty of Medicine, University of Debrecen, Debrecen, Hungary

**Keywords:** SPECT, SPECT/CT, Multi-pinhole, Nuclear cardiology, Image quality

## Abstract

**Background:**

In this study we evaluated the imaging capabilities of a novel Multi-pinhole collimator (MPH-Cardiac) specially designed for nuclear cardiology imaging on a Triple-NaI-detector based SPECT/CT system.

**Methods:**

^99m^Tc point source measurements covering the field of view (FOV) were used to determine tomographic sensitivity (TS_pointsource_) and spatial resolution. Organ-size tomographic sensitivity (TS_organ_) was measured with a left ventricle (LV) phantom filled with typical myocardial activity of a patient scan. Reconstructed image uniformity was measured with a 140 mm diameter uniform cylinder phantom. Using the LV phantom once filled with ^99m^Tc and after with ^123^I, Contrast-to-noise ratio (CNR) was measured on the reconstructed images by ROI analysis on the myocardium activity and on the LV cavity. Furthermore, a polar map analysis was performed determining Spill-Over-Ratio in water (SOR_water_) and image noise. The results were compared with that of a dual-head parallel-hole low energy high resolution (LEHR) collimator system. A patient with suspected coronary artery disease (CAD) was scanned on the LEHR system using local protocol of 16 min total acquisition time, followed by a 4-min MPH-Cardiac scan.

**Results:**

Peak TS_pointsource_ was found to be 1013 cps/MBq in the axial center of the FOV while it was decreasing toward the radial edges. TS_organ_ in the CFOV was found to be 134 cps/MBq and 700 cps/MBq for the LEHR and MPH-Cardiac, respectively. Average spatial resolution throughout the FOV was 4.38 mm FWHM for the MPH-Cardiac collimator. Reconstructed image uniformity values were found to be 0.292% versus 0.214% for the LEHR and MPH-Cardiac measurements, respectively. CNR was found to be higher in case of MPH-Cardiac than for LEHR in case of ^99m^Tc (15.5 vs. 11.7) as well as for ^123^I (13.5 vs. 8.3). SOR_water_ values were found to be 28.83% and 21.1% for the ^99m^Tc measurements, and 31.44% and 24.33% for the ^123^I measurements for LEHR and MPH-Cardiac, respectively. Pixel noise of the ^99m^Tc polar maps resulted in values of 0.38% and 0.24% and of the ^123^I polar maps 0.62% and 0.21% for LEHR and MPH-Cardiac, respectively. Visually interpreting the patient scan images, MPH-Cardiac resulted in better image contrast compared to the LEHR technique with four times shorter scan duration.

**Conclusions:**

The significant image quality improvement achieved with dedicated MPH-Cardiac collimator on triple head SPECT/CT system paves the way for short acquisition and low-dose cardiovascular SPECT applications.

## Background

Single Photon Emission Computed Tomography (SPECT) solutions for Myocardial Perfusion Imaging (MPI) were used for almost four decades as diagnostic tools for patients with known or suspected coronary artery disease (CAD) [1, 2]. The visual interpretation of SPECT images along with image-derived quantitative measures (e.g., summed scores or total perfusion deficit (TPD)) [3, 4] provide high sensitivity diagnosis of the extent and severity of ischemia. Beyond perfusion, ^123^I-metaiodobensylguanidine (^123^I-*m*IBG) SPECT was also effectively introduced for the molecular level diagnosis of innervation disorders related to cardiomyopathies [5–8] as well as accurate prognosis [9] and clinical decision making [10] in case of patients with chronic heart failure (HF). While these clinically proven applications are widely used, the image quality of conventional NaI(Tl) dual-head SPECT with parallel-hole collimators remained limited due to the relatively low tomographic sensitivity and spatial resolution of this technique. During a conventional SPECT scan, only a few parts-per-million of the injected radiopharmaceutical is detected. Attempting to overcome this limitation several scanner design concepts were introduced, including heart-focused astigmatic collimators convergent on the image center [11–13] or organ-specific dedicated multi-pinhole (MPH) SPECT collimators [14–16] on Sodium Iodide (NaI(Tl)) crystal detectors, or the recently widely emerging solid-state Cadmium-Zinc-Telluride (CZT) detector-based heart-focused dedicated cardiac SPECT systems [17–19]. In addition, it is worth mentioning the Alcyone aperture approach, which is a combination of an MPH collimator focused on the heart with CZT technology in case of the MyoSPECT and the Discovery NM 530c systems [20–22]. All of the above-mentioned approaches can provide four to seven times higher tomographic sensitivity at the myocardium region compared to conventional SPECT imaging. The major clinical impact of myocardial SPECT sensitivity improvement is that it paves the way for shorter acquisition times and consequently lower probability of patient motion. Furthermore, it can also offer imaging protocols with reduced dose but uncompromised image quality. Moreover, these SPECT systems may provide a widely available opportunity to measure quantitative parameters from first-pass dynamic SPECT, such as absolute myocardial blood flow (aMBF) and myocardial flow reserve (MFR) which are proven prognostic markers improving risk stratification of CAD patients [23–25].

While the diagnostic and prognostic advantages of dedicated cardiac SPECT systems are already well reported [1, 2, 16–18, 23, 26], the clinical use of general-purpose SPECT systems equipped with cardiac specific collimators is still very relevant [2, 15, 16, 27]. In case of the heart-focused collimators the imaging performance [11, 13, 28, 29] along with the accurate clinical use [12, 30–34] has been already thoroughly investigated. Furthermore, the image quality performance of these systems was compared with CZT based and conventional SPECT using both phantom and patient scans [35]. MPH collimators specifically designed for SPECT imaging of the heart were also introduced [36, 37], featuring nine [38], twenty [39] or a combination of sixteen and twenty [40] pinholes for dual-detector SPECT as well as six pinhole collimators for a triple-detector system [41]. These approaches revealed better tomographic sensitivity and image quality performance compared to conventional low energy high resolution (LEHR) collimator NaI(Tl) SPECT. In this study we assessed the imaging performance of a new MPH collimator technology featuring thirty-six pinholes specifically designed for nuclear cardiology examinations on a triple-NaI-detector-based SPECT/CT system [42, 43]. We evaluated tomographic sensitivity, spatial resolution, reconstructed image uniformity, image quality phantom measurements and a proof-of-principle patient scan. Where applicable, we compared these results with that of a conventional LEHR collimator-based dual-head SPECT/CT system. In case of the image quality measurements, both ^99m^Tc and ^123^I were used to evaluate the performance with these isotopes to ensure appropriate perfusion and innervation imaging applications.

## Materials and methods

### Triple-NaI-detector SPECT system with multi-pinhole collimator

**Fig. 1:**
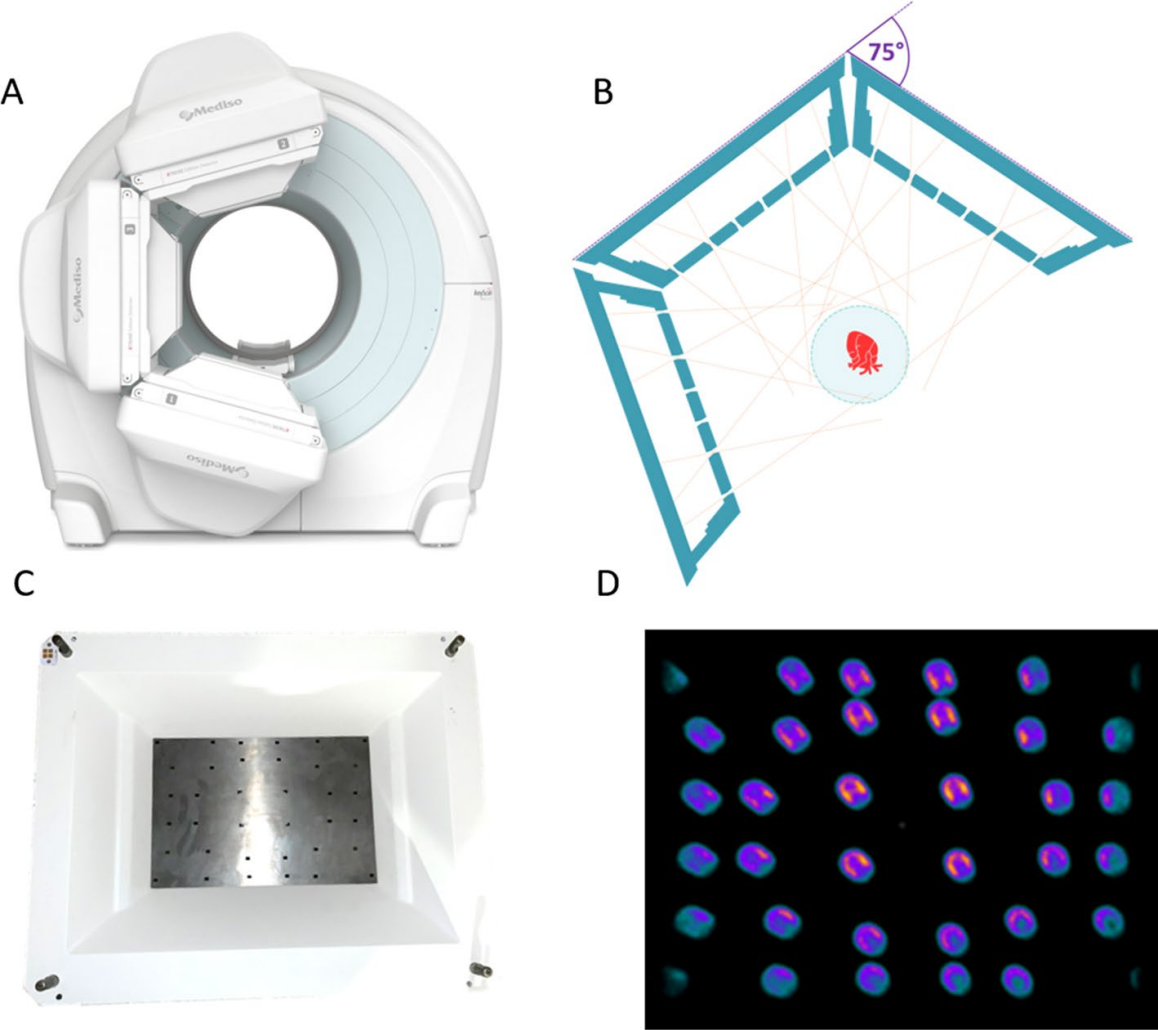
C-shape detector configuration of the triple-NaI-detector SPECT/CT system with MPH-Cardiac collimators (**A**); Schematic figure of projection trajectories of myocardium activity distribution for the MPH-Cardiac collimator system (**B**); A photograph of the rear view of the MPH-Cardiac collimator (**C**); Single projection image of the anthropomorphic phantom LV insert filled with ^99m^Tc (**D**)

A general-purpose triple-NaI-detector SPECT/CT system, the AnyScan^®^ TRIO (Mediso Medical Imaging Systems, Budapest, Hungary) was used in this study (Fig. 1. Panel A). Each SPECT detector head includes a 3/8″ NaI(Tl) scintillation crystal with 593 mm (transaxial) × 470 mm (axial) dimensions. Each detector head includes 94 photomultiplier tubes (PMTs) providing 2.9 mm UFOV intrinsic resolution of the detector, which is important for precise reconstruction since MPH is minifying projections on the detector surface. MPH-Cardiac collimator was designed for dedicated SPECT imaging with high count sensitivity at the location of the left ventricle (LV). The collimator features an 18 mm thick solid tungsten aperture plate including thirty-six pinholes (Fig. 1. Panel D). They are arranged to maximize count sensitivity and avoid multiplexing artifacts in the myocardium region [42, 44, 45] (Fig. 1. Panel C, D and E). The SPECT/CT system features a lateral-direction motion of the patient bed, allowing to position the myocardium in the rotational center of all three detector heads. Furthermore, the step-and-shoot detector movement is synchronized with table translation during the image acquisition process providing a helical scan trajectory. The MPH-Cardiac collimator system was designed to focus on the heart at maximum detector radius, avoiding collisions due to patient body proximity to the detector surface. In addition, a pressure sensitive touch foil is used for crash prevention on the surface of the MPH-Cardiac collimator and on the sides of the pyramids. Comparison measurements were taken with a general-purpose dual-head SPECT/CT system and LEHR collimators, the AnyScan^®^ SC (Mediso Medical Imaging Systems, Budapest, Hungary) was used in this study. Each SPECT detector head is including 3/8″ NaI(Tl) scintillation crystal with 593 mm (transaxial) × 470 mm (axial) dimensions and 60 PMTs, providing 3.3 mm UFOV intrinsic resolution of the detector.

### Image acquisition and reconstruction settings

Image acquisitions were performed with LEHR collimators on a dual-detector head SPECT system in 90° detector configuration, 64 views covering 180° scan arc, and the MPH-Cardiac collimators on a triple-NaI-detector SPECT system (AnyScan TRIO) in 75° detector configuration rotating 225° scan arc, 40 mm table movement helical scan with 24 gantry rotation steps. Helical scan means 40 mm in total table movement throughout the scan with 40/24 mm table movement in each step. Acquisition scan duration was 16 min in case of the LEHR and 4 min in case of the MPH-Cardiac measurements. These are net acquisition times excluding gantry rotation and table movement. Image reconstructions were performed with Tera-Tomo™ 3D SPECT-Q using the following settings: 8 iterations 4 subsets 3.3 mm voxel size in case of the LEHR measurements, 24 iterations 3 subsets 3.6 mm voxel size for the MPH-Cardiac measurements. In all cases we used vendor specific scatter correction and CT-based attenuation correction. The scatter of gamma rays in both the human tissue and the material of the collimator is modeled in the reconstruction software including changes in the energy of the photons. Only those counts are used for the image reconstruction, which fall into the predefined energy window in the acquisition protocol. In case of ^123^I, the reconstruction simulates more than one gamma photons simultaneously from the same isotope location, to more accurately model the actual emission, scattering, penetration in the edge of the pinholes as well as in the body of the patient. A more detailed description of the Tera-Tomo™ reconstruction method is described in a previous work [46]. A Gaussian post-filter was applied with 3 kernels and 4 sigma on both image sets. Any alteration from these acquisitions or reconstructions are indicated in the sections below.

### Tomographic sensitivity

Two types of tomographic sensitivity measurements were performed: first using a point source prepared in a small Eppendorf tube to represent the point-by-point sensitivity profile (TS_pointsource_), and secondly a clinically more relevant organ-specific tomographic sensitivity (TS_organ_) using a cardiac LV insert phantom. A 5 µl point source of 20.5 MBq ^99m^Tc was prepared and measured throughout the central axis of the field of view (FOV) with 1 cm steps. This measurement series were repeated at 5 cm, and 10 cm transaxial offsets with 2 cm steps using the same point source. Radial sensitivity profile was measured with 2 cm steps using the same point source. Each measurement was taken with acquisition settings described above, but with no table movement and 120 s time duration. All measurements were decay corrected and tomographic sensitivity (T*S*_*pointsource.*_) was calculated based on the accumulated counts according to Eq. 1:

1 $${\text{TS}}_{{{\text{point}}\;{\text{source}}}} = \frac{{{\raise0.7ex\hbox{${{\text{Accumulated}}\;{\text{ Counts}}}$} \!\mathord{\left/ {\vphantom {{{\text{Accumulated}}\;{\text{ Counts}}} {T_{{{\text{acq}}}} }}}\right.\kern-0pt} \!\lower0.7ex\hbox{${T_{{{\text{acq}}}} }$}}}}{{A_{{{\text{sst}}{.}}} }}$$

where *A*_sst._ refers to source activity at scan start, *T*_acq_. refers to acquisition time duration. Tomographic organ sensitivity (TS_organ_) was determined using the ^99m^Tc filled DataSpectrum LV phantom measurements and image reconstructions described below. A large, 150 mm diameter VOI was applied on reconstructed SPECT images including the entire myocardium phantom to determine the accumulated counts. Using the total accumulated counts, the filled radioactivity and net acquisition time of these measurements, the TS_organ_ was calculated as proposed by Imbert et al.[35] as follows:

2 $${\text{TS}}_{{{\text{organ}}}} = \frac{{{\text{Accumulated}} \;{\text{counts}}}}{{T_{{{\text{acq}}}} \times A_{{{\text{lv}}}} }}$$

where A_lv_ refers the activity in the left ventricle in MBq, T_acq_ refers to acquisition time duration in seconds.

### Spatial resolution

Spatial resolution was determined in terms of Full Width at Half Maximum (FWHM) and Full Width at Tenth Maximum (FWTM) using the acquired image data for point source tomographic sensitivity. Point source images were reconstructed using Tera-Tomo™ 3D SPECT-Q with the settings and post filters described above, except for using 1.8 mm voxel size. ImageJ NMQC plugin [47] was used to calculate FWHM and FWTM on each reconstructed image set. Mean and Standard Deviation (St.Dev.) in axial, transaxial and radial directions were calculated for all axial and radial measurement series, as well as the absolute average spatial resolution of all point-source measurements.

### Reconstructed image uniformity

A uniform cylinder phantom with 140 mm diameter [48], 105 mm length and 1200 ml total volume was filled with 16.32 ± 1.64 MBq ^99m^Tc at the start of the acquisition. Two different imaging modes were used as described above along with the defined image reconstruction settings and post filters. A spherical volume of interest (VOI) with 50 mm diameter was applied on the reconstructed images to investigate reconstructed image noise. Mean and St.Dev. values were calculated on voxels within the applied VOIs.

### Spill-over-ratio and noise analysis on polar maps

The commercially available LV insert phantom (Data Spectrum Corporation, Hillsborough, NC, USA) that mimics a perfusion deficit was used to carry out image quality comparison based on a myocardium shape activity distribution. The cold deficit chamber was filled with water. The LV walls for the first set of measurements were filled with a solution containing 10.00 ± 0.53 MBq ^99m^Tc, and for the second set of measurements with 8.50 ± 0.26 MBq ^123^I. Image acquisitions of 16 min were performed for the LEHR, while in case of the MPH-Cardiac measurement a 4-min dataset was generated from the list-mode data of the 16 min measurements. The same single energy window was applied for LEHR and MPH-Cardiac when using the same isotope. In case of ^123^I the lower and upper limits of this window was set to ± 10% from the primary peak of 159 keV. For the ^99m^Tc measurements, the applied energy window was ± 10% from the primary peak of 140 keV. Image reconstructions were performed with Tera-Tomo™ 3D SPECT-Q using the settings and post filters described above. Contrast-to-noise ratio (CNR) was measured on the reconstructed images of all cases by applying a half-moon shaped ROIs on myocardium activity and circular ROIs on the LV cavity as indicated in Fig. 4 and calculating CNR as:

3 $$\frac{{\left( {M_{{{\text{Myo}}}} + M_{{{\text{LVcav}}{.}}} } \right)}}{{\sqrt {{\text{SD}}_{{{\text{Myo}}}}^{2} + {\text{SD}}_{{{\text{LVcav}}{.}}}^{2} } }}$$

where M_Myo_ refers to the mean of the myocardium ROI, M_LVcav_. is the mean of the LV cavity ROI, SD_Myo_ refers to the standard deviation of the myocardium ROI, SD_LVcav._ is the standard deviation of the LV cavity ROI. Perfusion polar maps were generated using InterView™ XP software (Mediso Medical Imaging Systems, Budapest, Hungary) from all LV phantom measurements. A large asymmetric manual ROI was placed on the uniform area, while an elliptical manual ROI on the deficit area of all four polar maps. Spill-Over-Ratio in water (SOR_water_) was calculated as mean value of the deficit ROI voxel values divided by mean value of the uniform ROI voxel values and multiplied by 100. Noise was calculated as square of the St.Dev. of the uniform ROI voxel values divided by mean value of the uniform ROI voxel values and multiplied by 100.

### Patient scan

A 67 years old male patient (BMI = 35,5) with suspected CAD was injected with 342 MBq ^99m^Tc-sestaMIBI. Pharmacological stress was performed using i.v. injection of 0.56 mg/kg dipyridamole. A 16 min acquisition was performed in supine position with a dual-head SPECT system using the local routine imaging protocol as described above, followed by a 4 min acquisition in supine position with the triple-NaI-detector SPECT using MPH-Cardiac with the imaging and reconstruction settings described above.

## Results

### Tomographic sensitivity

**Fig. 2:**
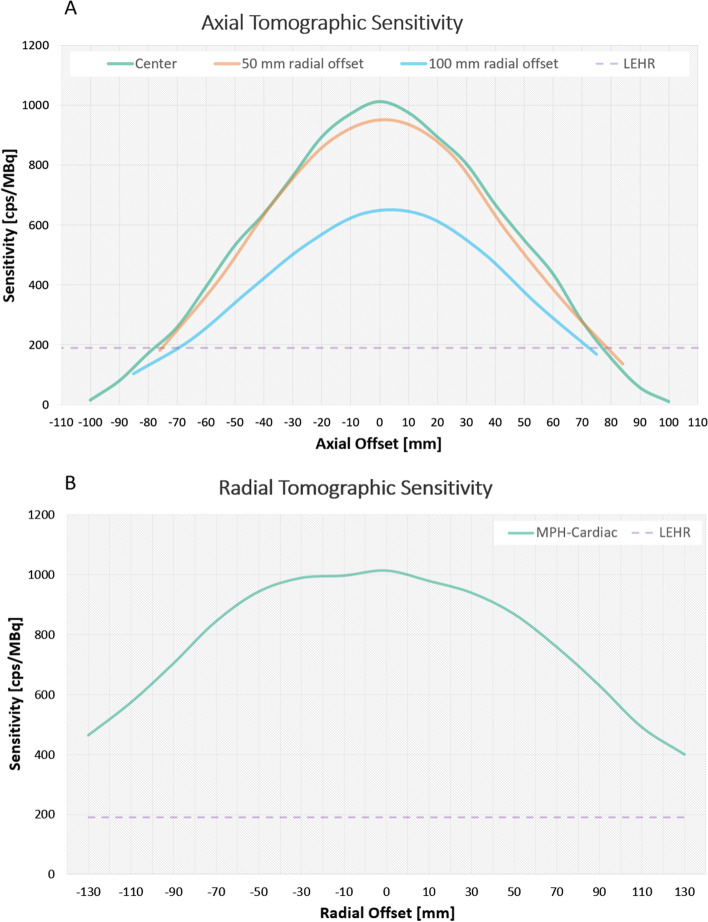
Axial (**A**) and Radial (**B**) tomographic sensitivity profiles of the MPH-Cardiac collimator system. The axial sensitivity profile was measured at 50 mm and 100 mm radial offsets. The reference line of the LEHR tomographic sensitivity is displayed as a dashed purple line

Peak tomographic sensitivity measured with the point source was found to be 1013 cps/MBq in the absolute axial center of the FOV (CFOV) while it is decreasing toward the radial edges: 951 cps/MBq at 5 cm radial offset from the CFOV and 641 cps/MBq at 10 cm radial from the CFOV. Meanwhile tomographic sensitivity decreases more drastically toward the axial edges of the FOV: 532 cps/MBq at 5 cm axial offset from the CFOV and 15 cps/MBq at 10 cm axial offset from the CFOV as can be depicted in Fig. 2. Panel A. TS_organ_ was found to be 134 cps/MBq versus 700 cps/MBq for the LEHR and MPH-Cardiac, respectively. Therefore, more than fivefold tomographic sensitivity gain can be achieved in the myocardium region with the MPH-Cardiac collimator and triple-NaI-detector SPECT.

### Spatial resolution

The absolute average spatial resolution was found to be 4.38 mm FWHM calculated from the measurements with the MPH-Cardiac collimators along the central axis of the FOV and through the centrum of the FOV in the radial directions. The separate axial and radial Mean FWHM results are presented in Table 1. Along with the St.Dev. values.

**Table 1:** Mean and Standard Deviation (St. Dev.) values of FWHM spatial resolution results calculated on the reconstructed images, measured along the central axis and through the centrum of the FOV in the radial directions

	Axial measurements FWHM [mm]			Radial measurements FWHM [mm]		
	X (axial)	Y (transaxial)	Z (radial)	X (axial)	Y (transaxial)	Z (radial)
Mean	4.91	4.85	3.36	4.85	4.21	4.10
St.Dev	0.35	0.47	0.72	0.49	0.67	0.53

### Image uniformity

**Fig. 3:**
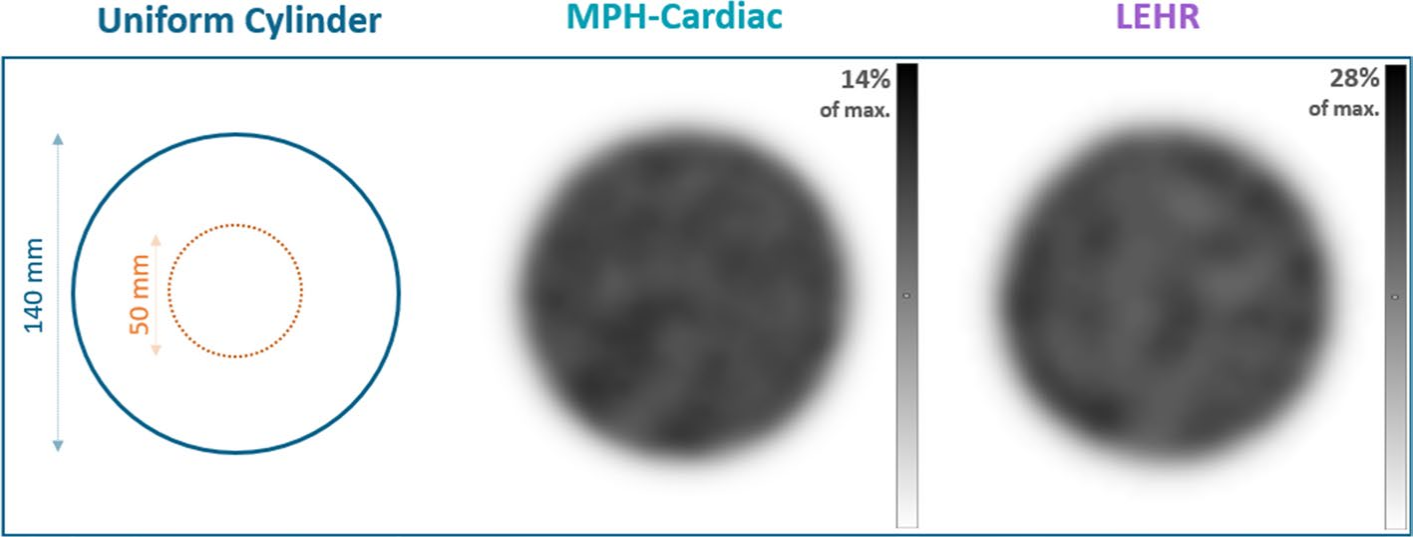
Schematic figure and real images of the uniform cylinder (140 mm diameter) filled with ^99m^Tc acquired on the AnyScan SC LEHR system and AnyScan TRIO with MPH-Cardiac collimator system. The upper limit of the inverted gray color bar compared to the maximum intensity in percentage is also indicated

Reconstructed SPECT images of the uniformity cylinder measurements can be seen in Fig. 3. The Mean and St.Dev values from the VOI analysis are presented in Table 2. The VOI analysis resulted 0.292% versus 0.214% uniformity values for the LEHR and MPH-Cardiac measurements, respectively.

**Table 2:** The table displays the measured Mean and St.Dev. accumulated count values within the applied 50 mm diameter VOI, and the calculated image uniformity

Collimator and Detector Configuration	50 mm diameter VOI Mean [counts]	50 mm diameter VOI St.Dev. [counts]	Image uniformity (%) ${100\times \left(\frac{St.Dev.}{Mean}\right)}^{2}$
LEHR 90°	37.55	2.03	0.292
MPH-CARDIAC 75°	287.16	13.29	0.214

### CNR, Spill-over-ratio and noise analysis

**Fig. 4:**
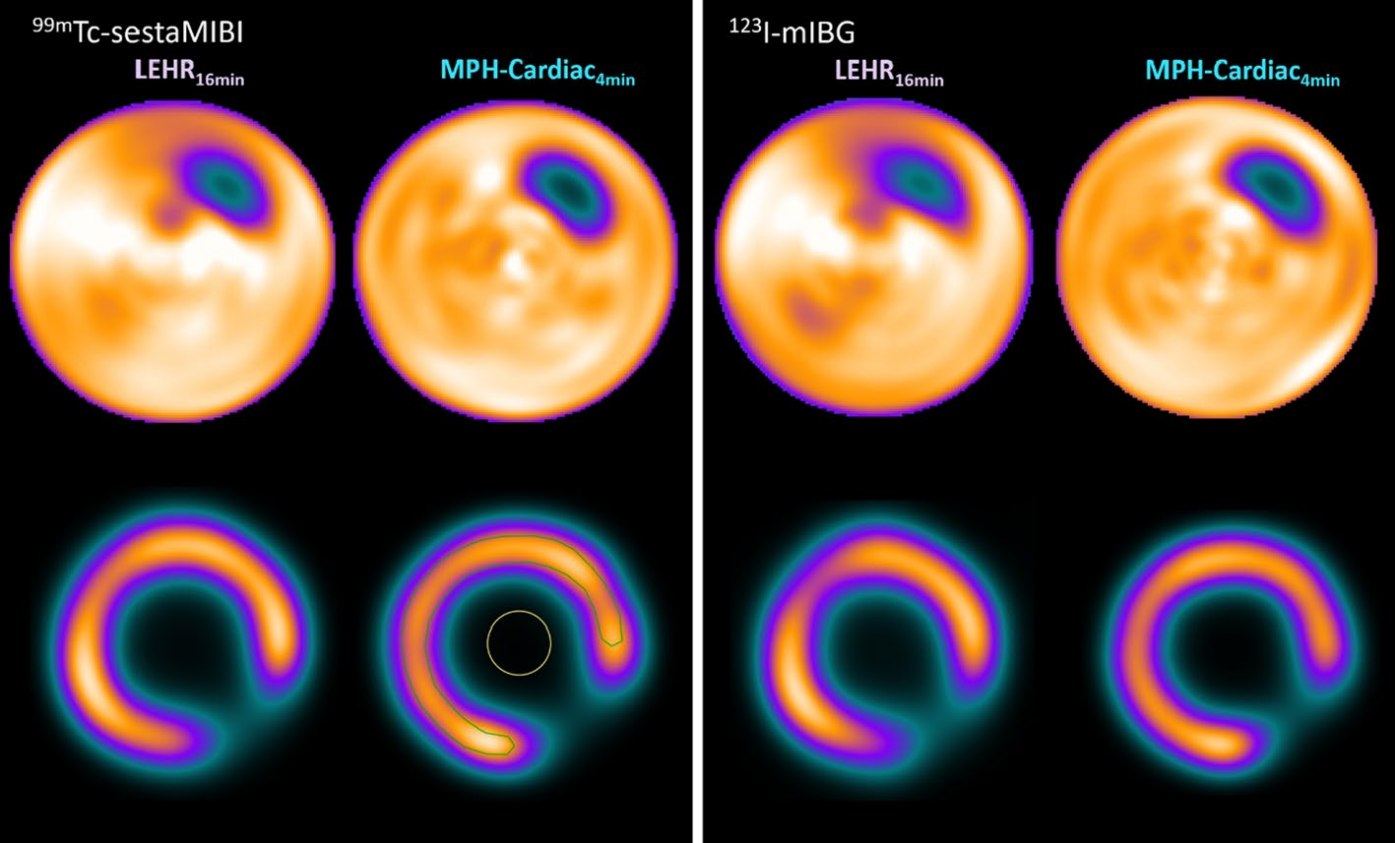
Polar Map representations of the Data Spectrum LV insert phantom filled with ^99m^Tc (left upper row) and ^123^I (right upper row) measured with the AnyScan SC with LEHR 16 min acquisition and the AnyScan TRIO with MPH-Cardiac collimator 4 min acquisition. Short axis images of the reconstructed LV inserts are presented for the ^99m^Tc (left lower row) and ^123^I (right lower row) measurements. Half-moon shaped ROI was applied on both the myocardium region of the phantom (green) and a circular ROI within the LV cavity (yellow). These ROIs were applied on all four LV SPECT images and CNR values were calculated based on Eq. 3

CNR was found to be 15.5 in case of MPH-Cardiac 11.7 for LEHR in case of ^99m^Tc, while it was 13.5 and 8.3, respectively, when using ^123^I. Polar map analysis was performed on the LV phantom measurements with ^99m^Tc and ^123^I. The MPH-Cardiac images showed much better contrast and image noise compared to the conventional LEHR technique as can be depicted in Fig. 4 and Table 3. Perfusion polar maps revealed SOR_water_ values of 28.8% versus 21.1% for the ^99m^Tc measurements with the LEHR and MPH-Cardiac configuration, respectively, as indicated in Table 3. In case of the ^123^I measurements, SOR_water_ values were found to be 31.4% versus 24.3% for the LEHR and MPH-Cardiac measurements, respectively. Pixel noise of the ^99m^Tc polar maps resulted values of 0.38% versus 0.24% for the LEHR and MPH-Cardiac, respectively. Meanwhile, ^123^I polar maps resulted values of 0.62% versus 0.21% for the LEHR and MPH-Cardiac, respectively. Visually interpreting the polar maps, in each case the MPH-Cardiac measurements resulted in better image contrast compared to the LEHR measurements for both isotopes despite of the shortened acquisition time as can be seen in Fig. 4.

**Table 3:** SOR and Noise values calculated from ROI analysis of reconstructed SPECT image Polar Maps generated from DataSpectrum LV insert filled with ^99m^Tc-sestaMIBI and ^123^I-mIBG measurements on the AnyScan TRIO MPH-Cardiac and AnyScan SC LEHR systems

Collimator and Clinical Detector Configuration	^123^I			^99m^Tc		
	CNR	SORwater [%]	Noise [%]	CNR	SORwater [%]	Noise [%]
LEHR 90° 16 min	8.3	31.4	0.62	11.7	28.8	0.38
MPH-CARDIAC 75° 4 min	13.5	24.3	0.21	15.5	21.1	0.24

### Patient scan

**Fig. 5:**
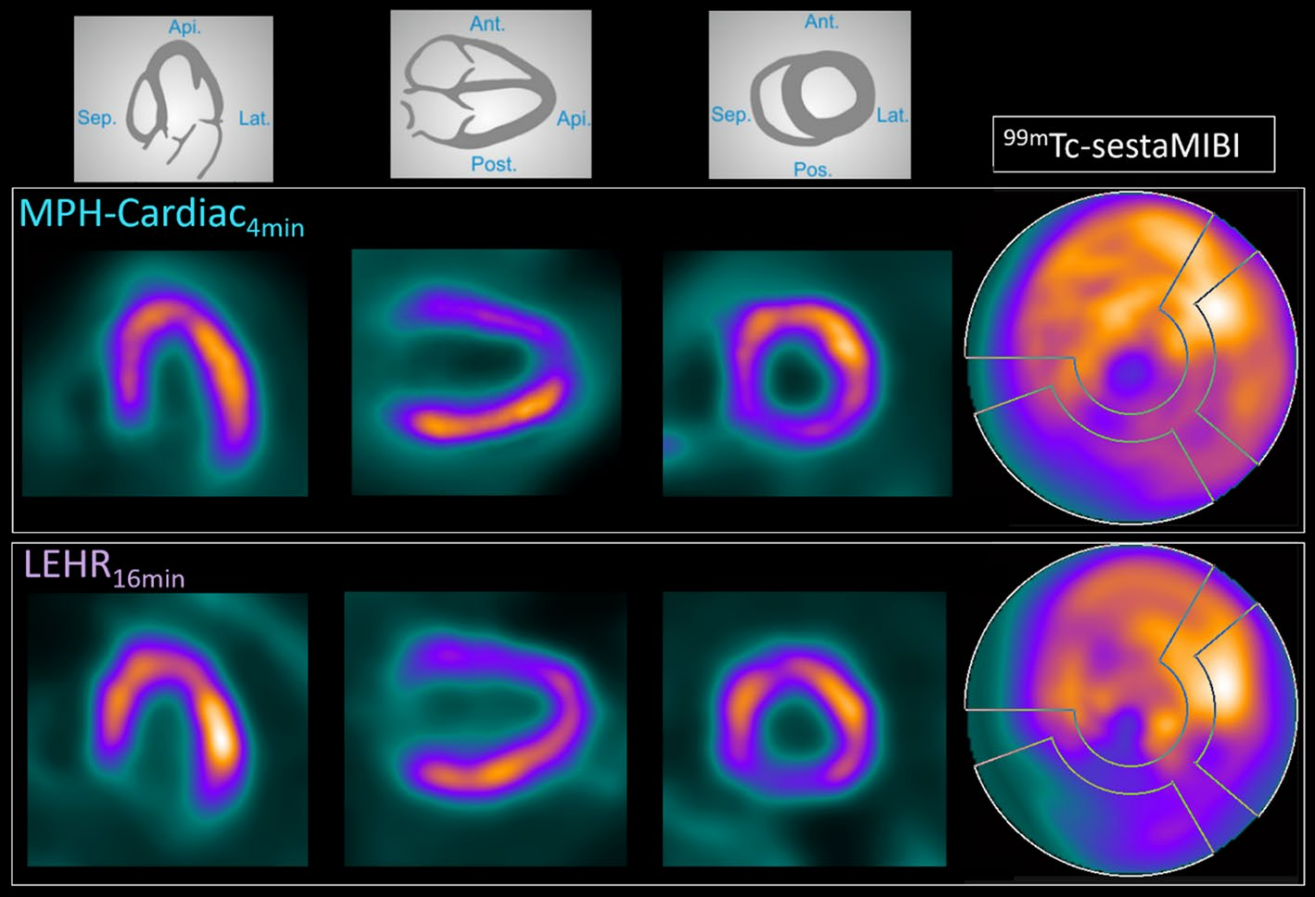
Representative post-stress SPECT image slices and polar maps of a 67 years old male CAD patient. Reconstructed images of the four minutes MPH-Cardiac acquisition (upper row) and sixteen minutes LEHR acquisition (lower row)

Reconstructed images and perfusion polar maps for the 16 min LEHR acquisition and 4 min MPH-Cardiac acquisition of the same patient are presented in Fig. 5. The reconstructed images revealed better contrast for MPH-Cardiac, as well as more homogenous polar map in the anterior wall region.

## Discussion

**Fig. 6:**
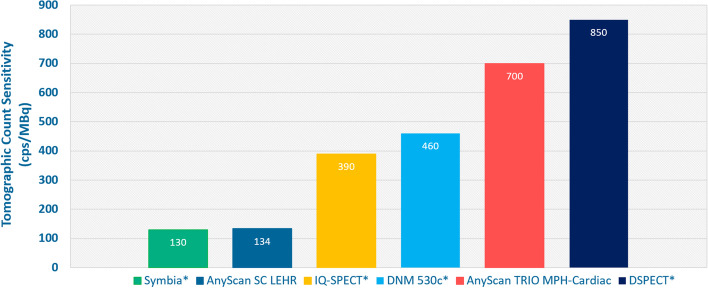
LV size tomographic count sensitivity (TSorgan) data measured on reconstructed images of the DataSpectrum LV phantom in case of AnyScan SC LEHR and AnyScan TRIO MPH-Cardiac, compared to several cardiac SPECT imaging systems of different vendors [35].*measured data from publication: Imbert et al. Journal of Nuclear Medicine 2012, 53:1987–1903; https://doi.org/10.2967/jnumed.112.107417

There is a well-known trade-off between spatial resolution and tomographic sensitivity in case of MPI with conventional SPECT collimators. Several scanner configurations have been evaluated in the last decades to overcome these limitations, providing high tomographic sensitivity in the myocardial region with uncompromised spatial resolution. Imaging performance and clinical relevance of high sensitivity CZT SPECT systems has been thoroughly reported [17–19, 26, 35, 49, 50], including pitfalls and artifacts [51] related to the specific imaging scheme of the heart in case of these dedicated cardiac scanners. CZT detectors have improved energy response compared to conventional NaI(Tl) crystals which significantly reduces scatter fraction of the measured data. CZTs also incorporate superior intrinsic resolution, however the high sensitivity characteristics of solid-state SPECT systems are often mistakenly attributed to the CZT crystal [17]. Indeed, the density of CZT is higher compared to NaI(Tl), however due to cost considerations the detectors are thinner, and therefore, the intrinsic detector efficiency is comparable to the conventional detectors. The overall improvement in tomographic sensitivity at the myocardial region is gained by the innovative, heart-focused scanner and collimator designs. While many dedicated cardiac SPECT systems are available with excellent imaging performance [1, 16–22, 50], the NaI(Tl) crystal based, large field of view general-purpose SPECT scanners can also provide high sensitivity solutions for MPI [15, 16]. Imaging performance of heart-focused astigmatic collimators on dual-head systems were thoroughly reported [11, 13, 28–30, 33, 34]. Furthermore, MPH collimators on a dual-detector or triple-detector SPECT scanners were also studied [14, 38–41] revealing the clinical potential of this concept. Improved sensitivity in the myocardium region provides shorter acquisition with lower probability of patient motion, imaging protocols with reduced dose, and offers the possibility to measure aMBF and MFR from first-pass dynamic SPECT. The AnyScan TRIO is a large field of view, Triple-NaI-detector-based Whole-Body SPECT system that can be easily transformed into a dedicated cardiac scanner with a simple exchange of LEHR and MPH-Cardiac collimators. The AnyScan TRIO was already studied with the dedicated MPH-Brain collimator for imaging performance in case of ^123^I-ioflupane DAT-scan [46] and the clinical accuracy of this dedicated brain imaging technique was also reported [52]. This current study summarizes the imaging performance of the AnyScan TRIO SPECT/CT with the MPH-Cardiac collimator system specially designed for nuclear cardiology imaging applications. The MPH-Cardiac collimator uses a similar projection method as the MPH-Brain, however, both the collimator pyramids, pinhole numbers (36 vs. 20) and the pattern of projections differ between the two techniques. The central pinholes of the MPH-Cardiac collimator represent an overlap-free sampling of the LV, therefore there will be no overlapping artifact when the final image is reconstructed. On the other hand, peripheral pinholes are focused both on the LV and the surrounding tissues. The central region would be underutilized if a stand-alone myocardium was imaged. In reality, activity uptake outside of the central FOV is present (in the liver, intestine, etc.). Therefore, a distortion-free reconstruction requests that enough independent and unambiguous measurements of the myocardium and its surroundings should be provided. This condition is satisfied using projections of both the central and the peripheral pinholes. In all cases where image reconstruction was applicable, we used the same settings as for the patient scan, except in case of the spatial resolution results where lower reconstructed voxel size of 1.8 mm was applied. Measurements revealed increased TS_pointsource_ of the triple-NaI-detector system with MPH-Cardiac up to 1013 cps/MBq compared to the conventional dual-detector LEHR sensitivity of 190 cps/MBq [46]. This increased sensitivity gain is attributed to both the MPH-Cardiac collimator design and the triple-NaI-detector in contrast to the parallel-hole and dual-detector conventional technique. In addition, this sensitivity gain is localized in the center of the FOV and decreasing toward the axial and radial edges, while for LEHR the sensitivity is relatively uniform throughout the entire FOV [46]. The limited useful FOV with high sensitivity gain demands cautious patient preparation to position the myocardium in the optimal FOV position, however, this phenomenon is also well reported for other heart-focused dedicated SPECT systems as well [11, 19, 51, 53]. The LV size tomographic sensitivity (i.e., TS_organ_) for the AnyScan TRIO MPH-Cardiac configuration resulted 700 cps/MBq, more than five folds higher than that of the LEHR dual-detector imaging. These results were lower than the count sensitivity results reported for the CZT-based D-SPECT system, while 1.5 times higher than that of the CZT-based Discovery NM 530 and 1.8 time higher than that of the NaI(Tl)-based focused collimator dual-detector IQ**-**SPECT system as it was reported by Imbert et al. [35], and as it is presented in Fig. 6.

Spatial resolution results are presented using the dedicated reconstruction method (Tera-Tomo™ 3D SPECT-Q) and not the gold standard Filtered Back Projection (FBP), since this method was not available for the MPH-Cardiac collimator system. However, this limitation is well reported for other dedicated SPECT systems as well [19, 48]. Both the visual assessment and the VOI analysis performed on the reconstructed images of uniform cylinder measurement confirmed that the triple-NaI-detector MPH-Cardiac technique provides better reconstructed image uniformity compared to the dual-detector LEHR technique. The applied activity for the DataSpectrum LV phantom ^99m^Tc measurements was comparable to the mean value of a large VOI placed on the myocardium region of four randomly chosen real CAD patient reconstructed images with mean injected activity of 377 MBq, resulting a mean reconstructed value of 11.4 MBq within the applied VOI. CNR was found to be better in case of MPH-Cardiac than for LEHR in case of ^99m^Tc (15.5 vs. 11.7) as well as for ^123^I (13.5 vs. 8.3). However, since many publications highlight the importance of summed difference scores (SDS) calculated on polar maps to diagnose reversible perfusion deficits, it is very important to evaluate the image quality not only on the reconstructed SPECT images but also on the polar maps. The LV phantom derived polar map analysis revealed that when using ^99m^Tc the SOR_water_ values were better in case of the MPH-Cardiac compared to LEHR 21.1 versus 28.8, respectively, while acquisition time was four times shorter (4 min). Polar map pixel uniformity was also superior for the MPH-Cardiac compared to LEHR: 0.24% and 0.38% regardless of the shortened acquisition time in case of MPH-Cardiac. Similarly, for the ^123^I isotope, SOR_water_ was found to be superior for MPH-Cardiac (24.3) compared to LEHR (31.4). Polar map pixel uniformity was better for MPH-Cardiac (0.21%) when comparing to LEHR (0.62%). Since the polar map analysis of the current study revealed similarly good SOR_water_ and pixel noise values for the ^123^I measurements as for the ^99m^Tc measurement, the MPH-Cardiac collimator design could provide good image quality results for clinically relevant [5] ^123^I-*m*IBG cardiac innervation applications as well. The MPH-Cardiac features a solid tungsten aperture plate, which minimizes the potential of penetration of high energy photons in case of ^123^I, originating from the high energy peaks above the primary 159 keV [54]. A representative CAD patient scan revealed that excellent image quality can be achieved with the novel multi-pinhole technique of the MPH-Cardiac collimator even in case of four times shorter acquisition time compared to the conventional technique. These results anticipate fast and low-dose routine clinical applications in the future. It is important to emphasize that the imaging performance of the MPH-Cardiac system should be always evaluated while considering the equipment together with the dedicated image reconstruction algorithm Tera-Tomo™ 3D SPECT-Q. Beyond all these promising results, this study has some limitations. While the widely accepted NEMA NU-1 measurements became gold standard in performance comparison of SPECT systems, it is hardly applicable for organ-specific dedicated cardiac SPECT devices, and therefore it was not relevant in the current study. However, it is need to be emphasized that MPH-Cardiac is just one imaging mode of the AnyScan TRIO system (beyond LEHR Whole-Body and MPH-Brain modes) and the NEMA performance is indeed relevant when using LEHR collimators in Whole-Body mode. Therefore, only reasonable imaging performance measurements were carried out on this system, some of them proposed by former publications [35, 48]. All of these measurements could be extended to have a broader view of the imaging characteristics of this novel MPH concept. We only performed LV phantom measurements without any scattering media or background activity. A more comprehensive study should include evaluation with background as well, similarly as reported by Zoccarato et al. [55]. We only performed ^123^I measurements in case of the LV insert phantom due to the limited availability of this isotope at our institution. In case of ^123^I we only used LEHR collimator for the dual-detector system, however some publications suggest medium energy collimators for this isotope, which was not evaluated in this study. Measurements with medium energy collimator may have a significant impact on the SOR results of the LV phantom measurements with ^123^I. A more comprehensive study should include the rest of the phantom measurements and a patient scan with ^123^I-*m*IBG to ensure appropriate image quality for cardiac innervation application. The tungsten aperture together with the unique feature of SPECT for differentiation of isotopes based on their photopeak energy holds the potential for dual isotope imaging in the future. The patient scan was performed only in supine position, not considering the well reported benefits of combined prone plus supine imaging [4, 56–58]. Furthermore, patient image comparison with other dedicated systems could be biased due to the effects of patient positioning on the evaluation of myocardial perfusion SPECT [34, 49, 59]. Ecg-gated SPECT and consequently the functional parameters, i.e., LV Ejection Fraction (LVEF) was not assessed and validated in this study, a further investigation should compare volume measurement accuracy of gated SPECT with the MPH-Cardiac. We did not use respiratory gating or any other motion correction techniques. Addition of these methods may improve the image quality and precision of the MPH-Cardiac technique. We included a single representative CAD patient MPH-Cardiac image set together with the conventional dual-detector LEHR SPECT images; however, a comprehensive clinical validation should include large population of patient images to analyze further the clinical relevance of the AnyScan TRIO MPH-Cardiac system.

## Conclusions

Significant image quality improvement can be achieved with a novel multi-pinhole technology, the MPH-Cardiac combined with triple-NaI-detector SPECT, when comparing to conventional parallel-hole LEHR collimator dual-detector SPECT. This improvement can be attributed to the increased tomographic sensitivity and uncompromised spatial resolution in the myocardium region, provided by the novel collimator design. Image quality improvement in MPH-Cardiac with triple-NaI-detector SPECT paves the way for shorter acquisition times and lower effective doses for both perfusion and innervation imaging applications in nuclear cardiology.

## Data Availability

The datasets used and analyzed during the current study are available from the corresponding author on reasonable request.

## Abbreviations

aMBF: Absolute myocardial blood flow
BMI: Body mass index
CAD: Coronary artery disease
CFOV: Central field of view
CNR: Contrast-to-noise ratio
cps: Counts per second
CT: Computer tomography
CZT: Cadmium zinc telluride
FOV: Field of view
FWHM: Full width at half maximum
FWTM: Full width at tenth maximum
HF: Heart failure
LEHR: Low energy high resolution
LV: Left ventricle
MBq: Mega becquerel
MFR: Myocardial flow reserve
MPH: Multi-pinhole
MPI: Myocardial perfusion imaging
NaI(Tl): Thallium doped sodium iodide
PET: Positron emission tomography
PMT: Photomultiplier tube
ROI: Region of interest
SORwater: Spill-over-ration for water
SPECT: Single photon emission computed tomography
St.Dev.: Standard deviation
Tacq: Acquisition time
TPD: Total perfusion deficit
TSorgan: Organ-size tomographic sensitivity
TSpointsource: Point source size tomographic sensitivity
VOI: Volume of interest
